# 多孔有机笼毛细管电色谱手性柱的制备及应用

**DOI:** 10.3724/SP.J.1123.2021.07004

**Published:** 2022-04-08

**Authors:** Wenyan JIA, Minghua TANG, Junhui ZHANG, Liming YUAN

**Affiliations:** 云南师范大学化学化工学院, 云南 昆明 650500; School of Chemistry and Chemical Engineering, Yunnan Normal University, Kunming 650500, China; 云南师范大学化学化工学院, 云南 昆明 650500; School of Chemistry and Chemical Engineering, Yunnan Normal University, Kunming 650500, China; 云南师范大学化学化工学院, 云南 昆明 650500; School of Chemistry and Chemical Engineering, Yunnan Normal University, Kunming 650500, China; 云南师范大学化学化工学院, 云南 昆明 650500; School of Chemistry and Chemical Engineering, Yunnan Normal University, Kunming 650500, China

**Keywords:** 毛细管电色谱, 手性分离, 手性化合物, 多孔有机笼, capillary electrochromatography (CEC), chiral separation, chiral compounds, porous organic cages (POCs)

## Abstract

多孔有机笼(POCs)是一种新型的具有稳定有序三维空腔结构的多孔材料。通过2-羟基-1,3,5-均苯三甲醛与1*R*,2*R*-1,2-二苯基乙二胺发生席夫碱的缩合反应,合成了一种具有羟基功能基团的单一手性POCs材料;将其均匀涂敷在毛细管壁上制成色谱柱,利用电色谱柱成功拆分了二氢黄酮、吡喹酮、萘普生和3,5-二硝基-*N*-(1-苯乙基)苯甲酰胺4种手性化合物。探究了分离电压、缓冲溶液浓度及其pH值等因素对手性拆分的影响,获得了4种手性物质在POCs色谱柱上的最佳拆分条件。实验研究表明,二氢黄酮、吡喹酮、萘普生和3,5-二硝基-*N*-(1-苯乙基)苯甲酰胺获得优化分离效果所需的工作电压分别为13、14、14和12 kV;二氢黄酮适宜Tris-H_3_PO_4_缓冲溶液浓度为0.075 mol/L,吡喹酮、萘普生和3,5-二硝基-*N*-(1-苯乙基)苯甲酰胺适宜Tris-H_3_PO_4_缓冲溶液浓度为0.100 mol/L; 4种手性物质得到最佳分离效果时的pH值均为3.51。二氢黄酮、吡喹酮、萘普生和3,5-二硝基-*N*-(1-苯乙基)苯甲酰胺均达到基线分离,分离度分别为2.99、2.10、2.58和3.59。该POCs色谱柱还成功拆分了*o*,*m*,*p*-碘苯胺、*o*,*m*,*p*-硝基苯胺两种位置异构体。该研究表明POCs手性电色谱柱具有良好的手性识别能力,是一种优秀的手性分离材料,具有很大的电色谱手性分离应用前景。

## Abstract (en)

Capillary electrochromatography for enantioseparation has received considerable research attention in the past decades, because it integrates the advantages of classical electrophoresis and modern micro-column separation. Chirality is a fundamental feature of compounds found in nature and is also a major concern in the modern pharmaceutical industry. Porous organic cages (POCs) are defined as a class of porous materials with permanent ordered three-dimensional cavity structures that are different from those of porous materials, such as zeolite, metal-organic frameworks, covalent organic frameworks, and mesoporous silica. POCs have good solubility in general organic solvents and can be used as a chromatographic stationary phase conveniently coated inside a standard capillary column. Homochiral POCs with hydroxyl groups on the cage molecules were synthesized by imine-linked condensation of 2-hydroxy-1,3,5-triformylbenzene with (1*R*,2*R*)-1,2-diphenylethylenediamine. The thus-synthesized POCs were characterized by nuclear magnetic resonance (NMR) spectroscopy, Fourier-transform infrared (FT-IR) spectroscopy, X-ray powder diffraction (XRD) analysis, etc. In the FT-IR spectra, the absorption peaks at 1602, 1489, and 1458 cm^-1^ were attributed to the C=C-H and C=C tensile vibrations in the benzene ring. The strong characteristic absorption peak at 1636 cm^-1^ was attributed to the imine bond (C=N) stretching, the two peaks at about 2900 cm^-1^ were attributed to C-H bond vibration, and the absorption peak at 3420 cm^-1^ was attributed to the O-H pulling vibration. In the XRD patterns, the powder diffraction peaks of the POCs were consistent with the simulated data. These results indicated that POCs were successfully synthesized. Thermogravimetric analysis was performed in the temperature range of 25-800 ℃ (10 ℃/min), and the POCs were found to be stable up to 380 ℃. Dichloromethane was used as solvent to uniformly coat POCs on the capillary wall to prepare an electrochromatography column. Joule heat generated in electrophoresis was negligible under the experiment condition used for the open-tubular column. Four chiral compounds, viz. dihydroflavone, praziquantel, naproxen, and 3,5-dinitro-*N*-(1-phenylethyl)benzamide, were used as test compounds, and the electrochromatography separation conditions were optimized such that the best separations were obtained. The voltage was applied to separate the selected enantiomers in the range of 10-20 kV . Considering the good separation and appropriate migration time simultaneously, applied voltages of 13 kV and 12 kV were recommended for dihydroflavones and 3,5-dinitro-*N*-(1-phenylethyl)benzamide, respectively, as well as 14 kV for praziquantel and naproxen. The concentration of the buffer solution for dihydroflavonoids was 0.075 mol/L, and those for praziquantel, naproxen, and 3,5-dinitro-*N*-(1-phenylethyl)benzamide were 0.100 mol/L. The pH was 3.51 for all four substances. Resolutions of 2.99, 2.10, 2.58, and 3.59 were achieved on a POC chiral column for dihydroflavonoids, praziquantel, naproxen, and 3,5-dinitro-*N*-(1-phenylethyl)benzamide, respectively. Two positional isomers, viz. *o*,*m*,*p*-nitrophenol and *o*,*m*,*p*-nitrophenilamine, were also successfully separated with 0.100 mol/L Tris-H_3_PO_4_ at pH 3.51. Therefore, the chiral electrochromatography column showed good chiral recognition ability and the POC is an excellent separation material with excellent application prospect in chiral electrochromatography.

手性分离一直都是分离分析研究的重点和难点^[1]^,手性分离在制药、食品、化学等领域被广泛应用。例如在药物方面^[2]^,用于临床的2000多种药品中,就有500多种为外消旋体,手性药物的构型与细胞中的受体相匹配极为关键,如果不匹配,可能会降低药效,甚至会产生相反的药理作用,对人体造成伤害,所以手性药物的拆分显得尤为重要。毛细管电泳具有高效率、低能耗、分离模式多样等优点,已经发展成为手性分离最具应用前景的方法之一。毛细管电色谱(CEC)是结合了毛细管电泳与高效液相色谱优点而发展起来的一种分离分析方法,色谱柱由外层涂敷着聚酰亚胺、内径为25~100 μm的熔融石英管制成。石英色谱柱具有电阻大、内径小、比表面积大等特点,使得CEC可以在微电流、高电压的环境下工作。与HPLC相比,其分析用样品量较少,分离效率更高,分析成本更低,故CEC被广泛运用于化学、环保、医药等领域^[3,4]^。

多孔有机笼(POCs)^[5,6]^是通过亚胺键、碳碳键、硼酸酯键等共价键连接、依靠分子间堆积组装而成的多孔材料。该多孔材料的形状一般为四面体、八面体、十二面体、球型。但是由于形成的多孔材料^[7,8]^中的亚胺键不稳定,易发生水解,尤其是在酸性或碱性环境下,因此大大限制了多孔材料的应用。为解决这一问题,Cooper等^[9]^通过一种合成策略,将多孔有机笼的顶点与含羰基的物质(如乙醛)结合在一起,以此来维持晶体的稳定性,使其孔隙大小不发生改变。

目前,构筑手性POCs材料主要有两种方法:利用金属离子或者金属簇与纯光学手性有机配体通过自组装堆积形成;通过诱导引入手性基团,以非手性有机模板作为桥连配体组装堆积合成^[10]^,用伯胺与醛或者酮发生席夫碱反应获得有机分子胺笼^[11,12]^,且胺笼上的氨基较为活泼,为后期衍生和将POCs功能化提供了很多可能性。

POCs是一种较为理想的手性固定相材料。2015年,Yuan课题组^[13]^将多孔有机分子笼CC3-R用作毛细管气相色谱手性固定相,证实了该材料对手性物质有良好的分离能力。Kewley等^[14]^报道了POCs在气相色谱(GC)和电色谱中的应用,证明了POCs是一种可靠的拆分材料。2019年有文献^[15]^报道将POCs用于液相色谱分离手性化合物,并取得了不错的分离效果。POCs在吸附、分离、传感和催化等方面也有许多潜在运用^[16]^,揭示POCs的广阔应用前景。

POCs与沸石、金属-有机骨架、共价有机骨架、介孔二氧化硅等多孔材料不同,POCs在大部分有机溶剂中都能溶解。本文将POCs用于毛细管电色谱固定相,涂敷在毛细管内壁制成色谱柱,通过拆分外消旋体、位置异构体来研究其分离性能。通过缓冲溶液的浓度、操作电压以及pH值的变化来探究其对4种手性药物拆分效果的影响。

## 1 实验部分

### 1.1 仪器和试剂

CL 1020高效毛细管电泳仪(北京华阳利民仪器有限公司); Bruker DXR 500 MHz核磁共振波谱仪(瑞士布鲁克公司); D/Max 2000粉末衍射仪(日本Rigaku公司); Vario EL Ⅲ有机化学元素分析仪(北京来亨科贸有限责任公司); SDT-650热重分析仪(美国TA仪器); XL 30 ESEM-TMP扫描电子显微镜(荷兰飞利浦公司);纯水器(英国Elga公司)。

三氟乙酸、1*R*,2*R*-1,2-二苯基乙二胺、硫脲、苯酚、六亚甲基四胺、氘代氯仿(CDCl_3_)、氘代二甲亚砜(DMSO-d_6_)购于上海Adamas试剂有限公司;二氯甲烷、甲醇、二甲亚砜(DMSO)、氢氧化钠、2-羟基-1,3,5-均苯三甲醛、三氯甲烷、盐酸和磷酸(H_3_PO_4_)均购于天津风船化学试剂有限公司;生物缓冲剂三羟基甲基氨基甲烷(Tris)购于北京Solarbio公司;吡喹酮、二氢黄酮、萘普生、3,5-二硝基-*N*-(1-苯乙基)苯甲酰胺均购于比利时Acros Organics公司;位置异构体:*o*,*m*,*p*-碘苯胺、*o*,*m*,*p*-硝基苯胺购于上海Aladdin试剂有限公司。实验所用试剂纯度均≥99%。

### 1.2 2-羟基-1,3,5-均苯三甲醛的合成

参考文献^[17]^方法合成2-羟基-1,3,5-均苯三甲醛。在N_2_保护下,称取2.96 g (0.028 mol)苯酚与8.6 g (0.061 mol)六亚甲基四胺,加入30 mL三氟乙酸,于130 ℃油浴中搅拌24 h。将反应升温至150 ℃搅拌2.5 h后冷却至120 ℃,加入50 mL 4 mol/LHCl,静置12 h后获得淡黄色粉末,依次用甲醇、1 mol/L HCl、二氯甲烷洗涤得到2-羟基-1,3,5-均苯三甲醛。

### 1.3 多孔有机笼的合成

根据文献^[18]^方法合成POCs。将0.171 g (0.0015 mol) 1*R*,2*R*-二苯基乙二胺充分溶解在30 mL DMSO与3 mL三氯甲烷的混合溶液中,缓慢加入0.178 g (0.001 mol) 2-羟基-1,3,5-均苯三甲醛,然后加入0.01 mL三氟乙酸,并将上述混合物在室温下搅拌14 d。获得的橙色固体进行真空干燥后即可得到目标粗产物POCs,为了得到高纯度产品,依次用三氯甲烷、乙醚洗涤。合成路线见图1。

**图1:**
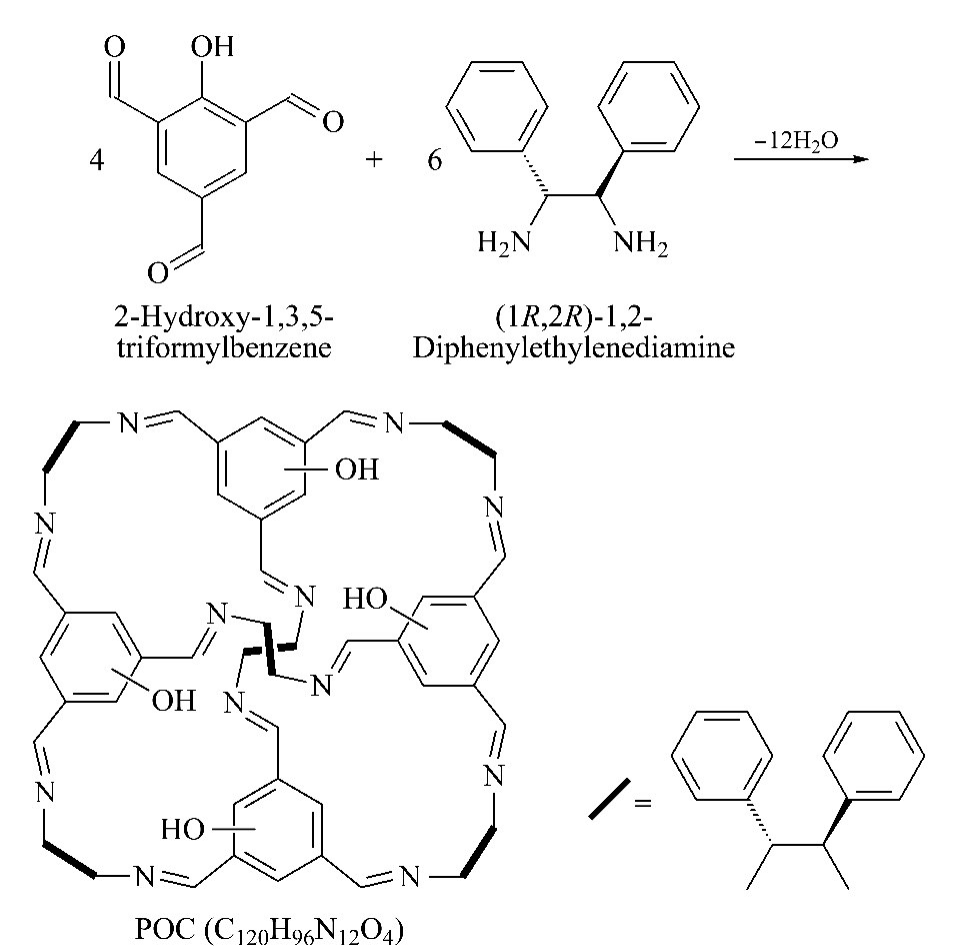
POCs的合成路线

### 1.4 多孔有机笼开管柱的制备

用1 mol/L NaOH溶液冲洗熔融石英毛细管2 h使其内壁粗糙化,接着用高纯水和0.1 mol/L HCl各冲洗1 h,再用高纯水冲洗至中性,最后将粗糙化后的石英毛细管柱用氮气在120 ℃下吹干、冷却后备用。

将POCs溶解于二氯甲烷溶液中,过滤。采用动态涂渍法^[19]^将该溶液在氮气的推动下通过上述内壁被粗糙化处理的毛细管,使其在管壁内形成一层湿的涂层,继续用氮气流将毛细管吹干,放入气相色谱柱温箱中进行老化,从30 ℃开始升温,以1 ℃/min的速率升至200 ℃,并在该温度下保持4 h,冷却后即得到所需的POCs开管柱。

### 1.5 毛细管电色谱实验条件

截取58 cm上述POCs开管柱(内径75 μm),在距末端8 cm处开窗,使该色谱柱的有效分离长度为50 cm。所用缓冲溶液均为Tris-H_3_PO_4_,电渗流标记物选用2 mg/mL硫脲。初次使用该色谱柱时,依次用去离子水、缓冲溶液冲洗色谱柱直至基线平稳。每次进样前,用缓冲溶液对色谱柱进行冲洗,以保证色谱柱的高效性与重复性。进样采用虹吸的方法,进样高差为5 cm,进样时间为2 s,样品的检测波长为254 nm。

拆分二氢黄酮、吡喹酮、萘普生和3,5-二硝基-*N*-(1-苯乙基)苯甲酰胺的工作电压分别为13、14、14和12 kV;二氢黄酮适宜缓冲溶液浓度为0.075 mol/L,吡喹酮、萘普生和3,5-二硝基-*N*-(1-苯乙基)苯甲酰胺为0.100 mol/L; 4种手性样品拆分时的pH值均为3.51。

## 2 结果与讨论

### 2.1 2-羟基-1,3,5-均苯三甲醛的结构表征

将2-羟基-1,3,5-均苯三甲醛溶解于DMSO进行核磁共振实验。^1^H NMR (500 MHz, DMSO-d_6_): *δ* 10.33 (s, 2H, OH), 10.02 (s, 1H, CHO), 8.56 (s, 2H, Ar-H);^13^C NMR (125 MHz, DMSO-d_6_): *δ* 192.14、191.18、166.74、137.77、128.58、124.75。上述所得数据与文献^[17]^报道一致,表明已经成功合成2-羟基-1,3,5-均苯三甲醛。

### 2.2 POCs的结构表征

采用核磁共振、红外光谱、X射线粉末衍射(XRD)等方法对合成的POCs进行表征。将POCs溶解于CDCl_3_中测得核磁共振氢谱,^1^H NMR (500 MHz, CDCl_3_): *δ* 8.80~8.56 (m, 4H), 8.38~8.25 (m, 4H), 8.10~8.03 (m, 4H), 7.94~7.86 (m, 8H), 3.46~3.24 (m, 12H), 1.83~1.46 (m, 48H),所得数据与文献^[18]^报道一致。

在红外光谱图2a中,1602、1489和1458 cm^-1^处的吸收峰由苯环中C=C-H和C=C拉伸振动产生。1636 cm^-1^处较强的特征吸收峰为亚胺键(C=N)拉伸带,位于2900 cm^-1^左右的两峰为C-H产生,位于3420 cm^-1^处的吸收峰是-OH拉伸带,所得实验数据与文献^[18]^报道结果一致。图2b是XRD测试图,POCs粉末衍射数据与模拟数据一致。以上测试均表明已经成功合成了POCs。

**图2:**
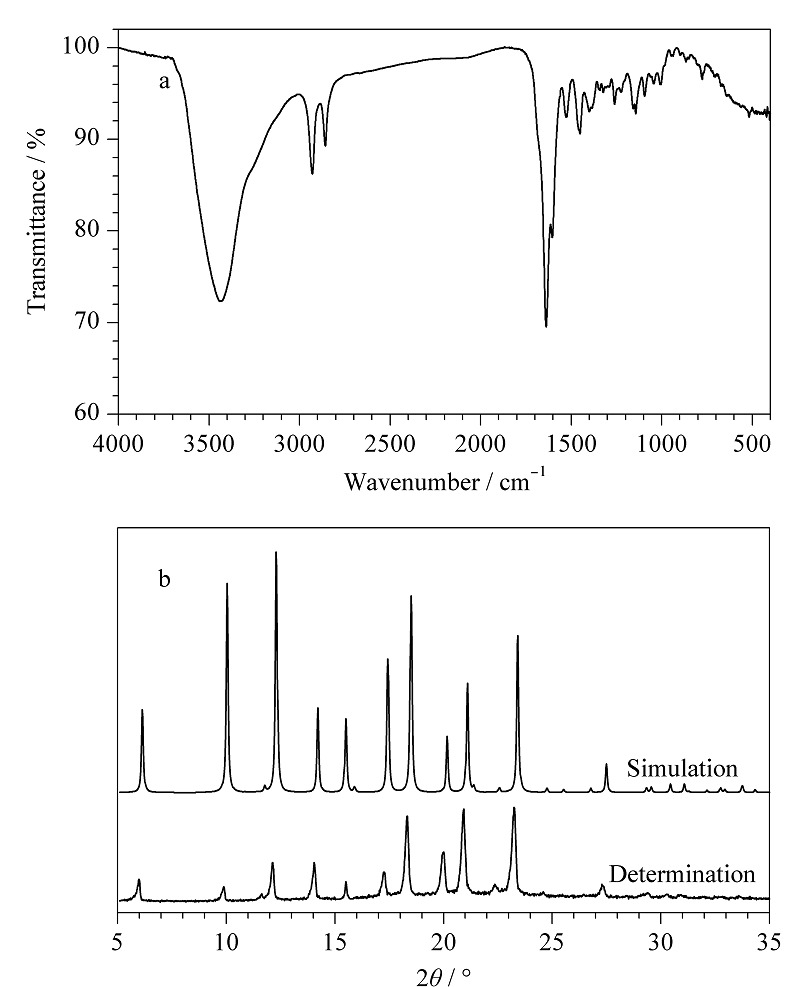
POCs的(a)红外光谱图和(b) X射线粉末衍射图

为考察该多孔有机笼的热稳定性,将热重仪的参数设置成以1 min/ ℃从室温升至800 ℃,进行热重分析,从图3可以看到,该材料在大约380 ℃时才开始分解,热稳定性好,电泳中产生的焦耳热对其稳定性没有影响,适于做手性开管毛细管柱。

**图3:**
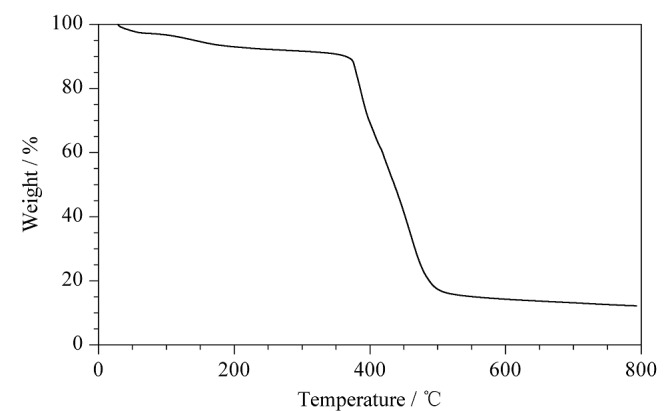
POCs热重曲线图

### 2.3 POCs的氮气吸附测试

该多孔有机笼是一种具有四面体结构的多孔材料,为测试其比表面积、空腔体积及孔径大小,进行氮气吸附实验,结果见图4。POCs的比表面积为209.91 m^2^/g,孔体积为0.078 cm^3^/g,孔径大小为1.98 nm。说明POCs比表面积较大,适用于一般有机化合物的分离。

**图4:**
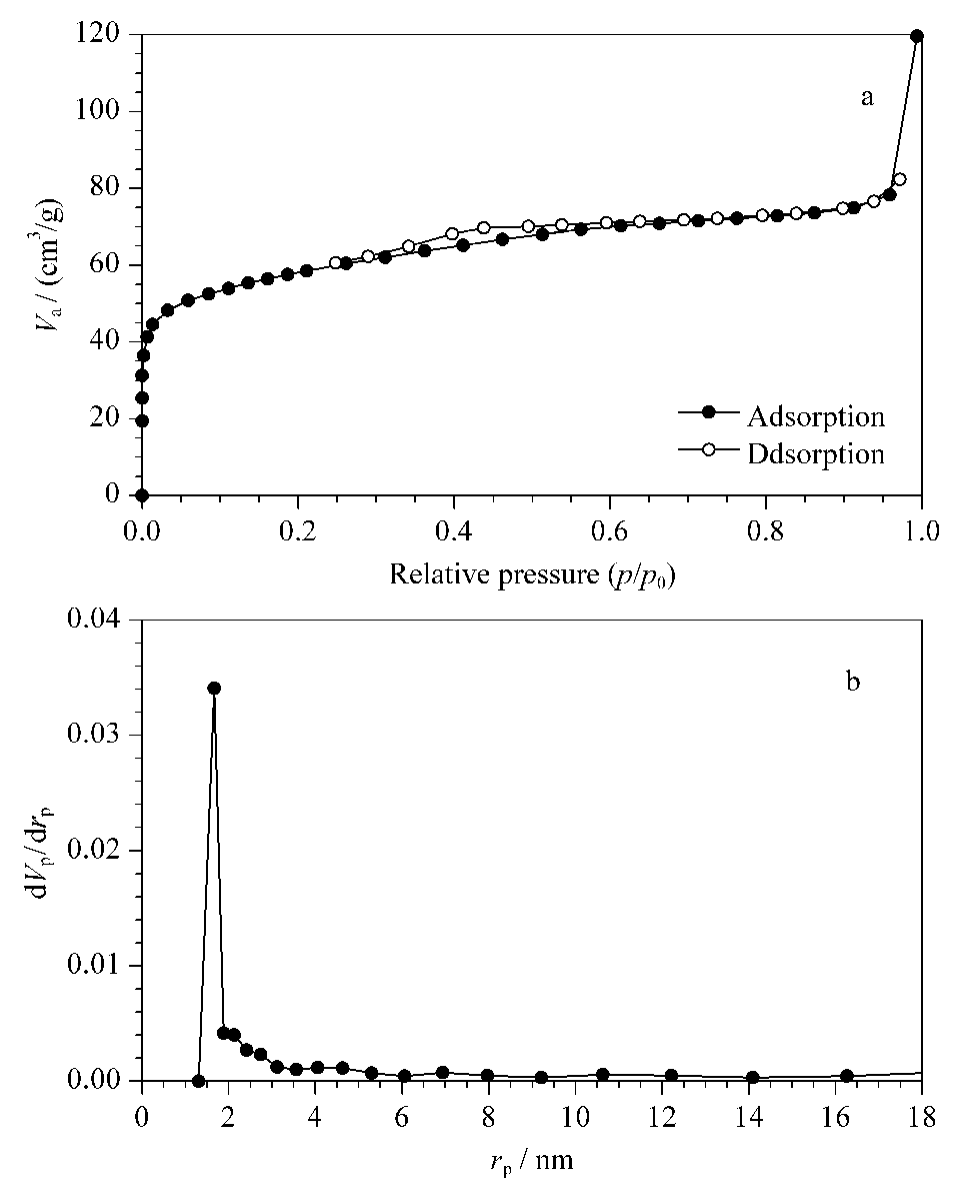
POCs的(a)氮气吸附脱附等温线和(b)孔径分布曲线

### 2.4 POCs毛细管柱扫描电镜表征

为观察POCs在毛细管内壁的涂渍情况,将制成的色谱柱进行扫描电镜分析(见图5),对比未涂敷的空毛细管柱与已经涂敷了POCs手性固定相的色谱柱扫描电镜图,可以明显看到毛细管内壁已经涂敷上一层厚度较为均匀的POCs材料。

**图5:**
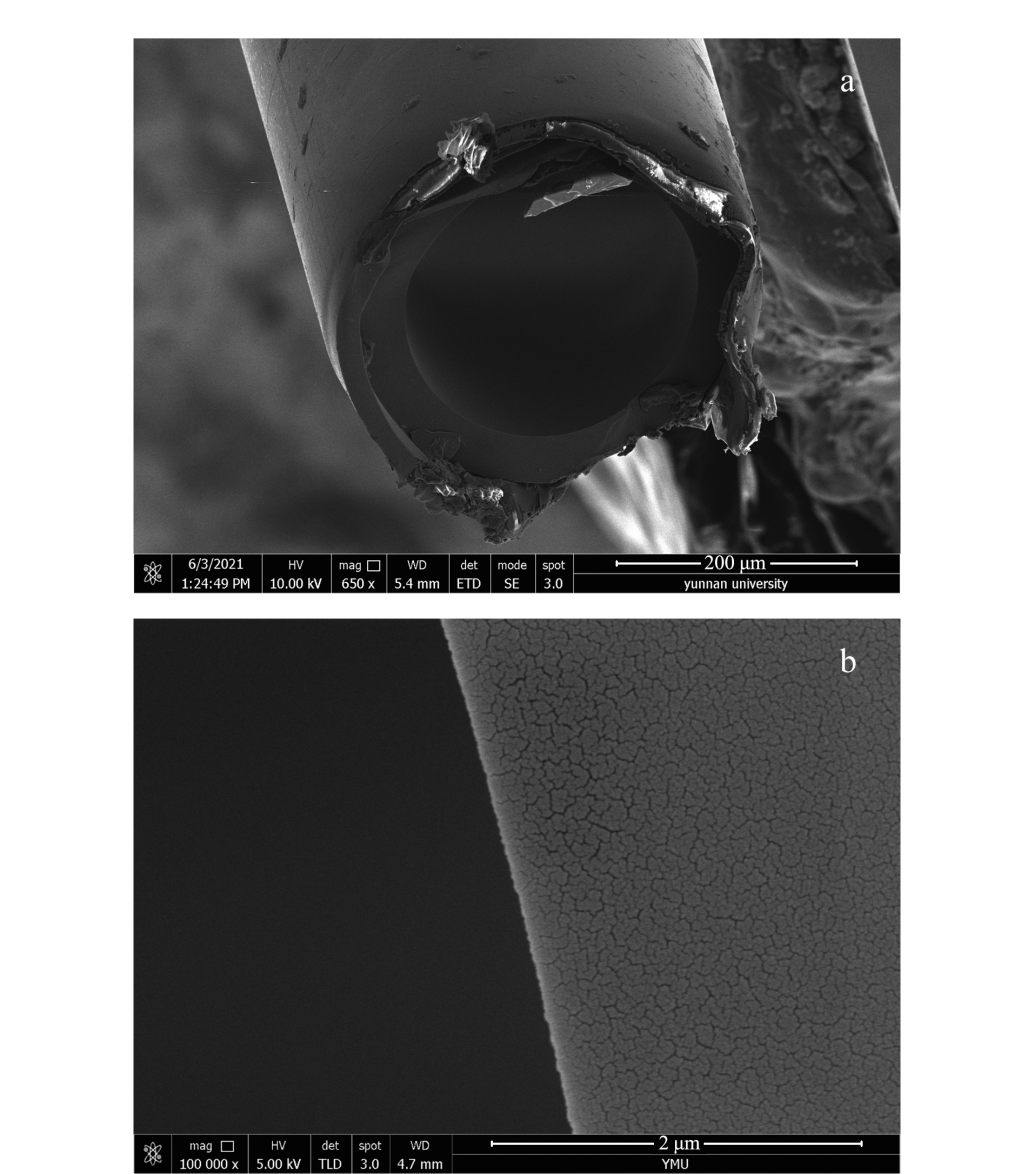
石英毛细管柱扫描电镜图

### 2.5 POCs色谱柱对手性化合物的拆分

POCs的手性识别能力,可能是受到该多孔有机笼独特的手性结构及官能团的影响。在弱范德华力的作用下,通过分子之间的自组装形成了具有四面体结构的多孔材料。当外消旋体进入到毛细管色谱柱中,与分子笼的表面或内部充分接触时,偶极-偶极之间的相互作用,以及*π-π*键、氢键等的相互作用会对手性分离产生影响。此外,较弱的范德华力也会产生一定影响。总之拆分手性分子是一个较为复杂的过程,涉及许多影响因素,但不可否认的是手性固定相在其中起到的作用是无可替代的。

为了探究该色谱柱对手性化合物的拆分能力,选用二氢黄酮、3,5-二硝基-*N*-(1-苯乙基)苯甲酰胺、萘普生和吡喹酮为样品,改变实验中的分离电压、缓冲溶液浓度、缓冲溶液pH值,优化拆分4种外消旋体的最佳实验条件。

2.5.1 分离电压对手性化合物拆分的影响

选用0.100 mol/L、pH 3.51的Tris-H_3_PO_4_缓冲体系,考察工作电压(10~20 kV)对分离对映异构体的影响。从表1部分数据可知,分离度(*R*_s_)先是随着电压的增加而增加,但随后分离度降低。增加操作电压,可以使电渗流增大,从而缩短样品出峰时间和增加分离度,但是过大的电流会加大焦耳热,降低化合物的分离度。二氢黄酮、3,5-二硝基-*N*-(1-苯乙基)苯甲酰胺、吡喹酮与萘普生获得较好分离效果所需的工作电压分别为13、12、14、14 kV。

**表1:** 不同分离电压下4种外消旋体的拆分结果

Sample	Separation voltage/kV	t_1_/min	t_2_/min	N_1_/(plates/m)	N_2_/(plates/m)	α	R_s_
Dihydroflavone	12	23.94	24.56	143995	151550	1.03	1.74
	13	19.01	19.77	138549	119962	1.04	2.49
	14	17.12	17.78	41422	44677	1.04	1.39
3,5-Dinitro-N-(1-phenylethyl)	11	23.96	24.28	196321	180938	1.01	1.02
benzamide	12	22.76	23.42	198603	151933	1.03	2.10
	13	15.82	16.24	141480	114148	1.03	1.65
Praziquantel	13	28.00	29.33	75144	59572	1.05	2.12
	14	26.40	28.02	63039	57193	1.06	2.58
	15	23.87	25.07	43719	32910	1.05	1.69
Naproxen	13	28.72	30.24	89250	32309	1.05	2.04
	14	24.31	26.32	63945	66397	1.08	3.59
	15	20.01	21.58	20083	53693	1.08	2.38

*N*: numbers of theoretical plates; *α*: separation factor; *R*_s_: resolution.

2.5.2 缓冲液浓度对手性化合物拆分的影响

在最优分离电压条件下,采用pH值为3.51但浓度不同的缓冲溶液,4种外消旋体的色谱拆分情况如表2所示。当分离二氢黄酮时,缓冲溶液浓度由0.050 mol/L升至0.075 mol/L,其分离度增大;继续增至0.125 mol/L,分离度却一直降低。当缓冲溶液浓度为0.05 mol/L时,3,5-二硝基-*N*-(1-苯乙基)苯甲酰胺和吡喹酮都无法分开,继续由0.075 mol/L增至0.100 mol/L时,3,5-二硝基-*N*-(1-苯乙基)苯甲酰胺、吡喹酮的分离度都增大,但是当浓度升至0.125 mol/L时,二者分离度都降低。当缓冲溶液浓度由0.050 mol/L增至0.100 mol/L时,萘普生的分离度逐渐增大,继续增至0.125 mol/L时,萘普生则无法分开。因此,适当范围内增加缓冲溶液的浓度可以提高手性分离效果,但过高浓度不但会降低拆分效率,还会导致不能手性分离。二氢黄酮得到较好分离的缓冲溶液浓度是0.075 mol/L,其他3种皆是0.100 mol/L。

**表2:** 不同浓度的缓冲溶液下4种外消旋体的拆分结果

Sample	c(Buffer)/(mol/L)	t_1_/min	t_2_/min	N_1_/(plates/m)	N_2_/(plates/m)	α	R_s_
Dihydroflavone	0.050	12.38	12.92	42454	82201	1.04	1.82
	0.075	17.03	18.22	60745	63857	1.07	2.99
	0.100	19.01	19.77	138549	119962	1.04	2.49
	0.125	21.04	21.52	148976	127893	0.82	1.23
3,5-Dinitro-N-(1-phenylethyl)	0.050	16.32	-	62347	-	-	-
benzamide	0.075	19.14	19.47	83864	72920	1.02	0.85
	0.100	22.76	23.42	198603	151933	1.03	2.10
	0.125	29.31	29.89	99048	85631	1.02	1.05
Praziquantel	0.050	18.13	-	38977	-	-	-
	0.075	22.36	23.10	40465	40944	1.03	1.16
	0.100	26.40	28.02	63039	57193	1.06	2.58
	0.125	29.61	30.36	70959	74600	1.03	1.20
Naproxen	0.050	15.59	16.01	154987	165741	0.79	2.47
	0.075	18.27	19.43	127973	144739	1.06	3.42
	0.100	24.31	26.32	63945	66397	1.08	3.59
	0.125	28.62	-	41085	-	-	-

-: not detected.

2.5.3 缓冲液pH值对手性化合物拆分的影响

在优化后的分离电压和缓冲液浓度下,考察实验缓冲溶液pH值对外消旋体分离的影响,实验数据如表3所示。当pH值从2.48增加到3.51,二氢黄酮、3,5-二硝基-*N*-(1-苯乙基)苯甲酰胺、吡喹酮分离度随之增大;当pH值增加到4.50时,分离度反而降低。萘普生pH值为3.51时,其分离度为3.59,但随着pH值的升高,反而无法将其分开。太低的pH值能影响色谱固定相的活性,太高的pH值能增大电渗流,缩短分析物的保留时间,影响分析物的拆分效果。拆分4种手性物质的最佳pH值均为3.51。

**表3:** 不同pH值缓冲溶液下4种外消旋体的拆分结果

Sample	pH	t_1_/min	t_2_/min	N_1_/(plates/m)	N_2_/(plates/m)	α	R_s_
Dihydroflavone	2.48	20.01	20.71	60856	89834	1.03	1.65
	3.51	17.03	18.22	60745	63857	1.07	2.99
	4.50	13.39	13.72	88291	52142	1.02	1.11
3,5-Dinitro-N-(1-phenylethyl)	2.48	29.64	30.38	38936	42591	1.02	0.88
benzamide	3.51	22.76	23.42	198603	151933	1.03	2.10
	4.50	18.02	18.23	81585	76079	1.01	0.58
Praziquantel	2.48	35.01	36.17	30253	43090	1.03	1.10
	3.51	26.40	28.02	63039	57193	1.06	2.58
	4.50	20.02	20.42	65693	68344	1.02	0.91
Naproxen	2.48	37.70	39.50	144608	168823	1.05	3.27
	3.51	24.31	26.32	63945	66397	1.08	3.59
	4.50	22.50	-	106038	-	-	-

经过上述3个实验的研究探讨,得到4种手性物质在最佳拆分条件的电色谱图(见图6),二氢黄酮、3,5-二硝基-*N*-(1-苯乙基)苯甲酰胺、吡喹酮与萘普生它们的分离度分别是2.99、2.10、2.58、3.59,全部都达到了基线分离,表明POCs手性色谱柱对外消旋体具有良好的手性识别能力和分离效果。

**图6:**
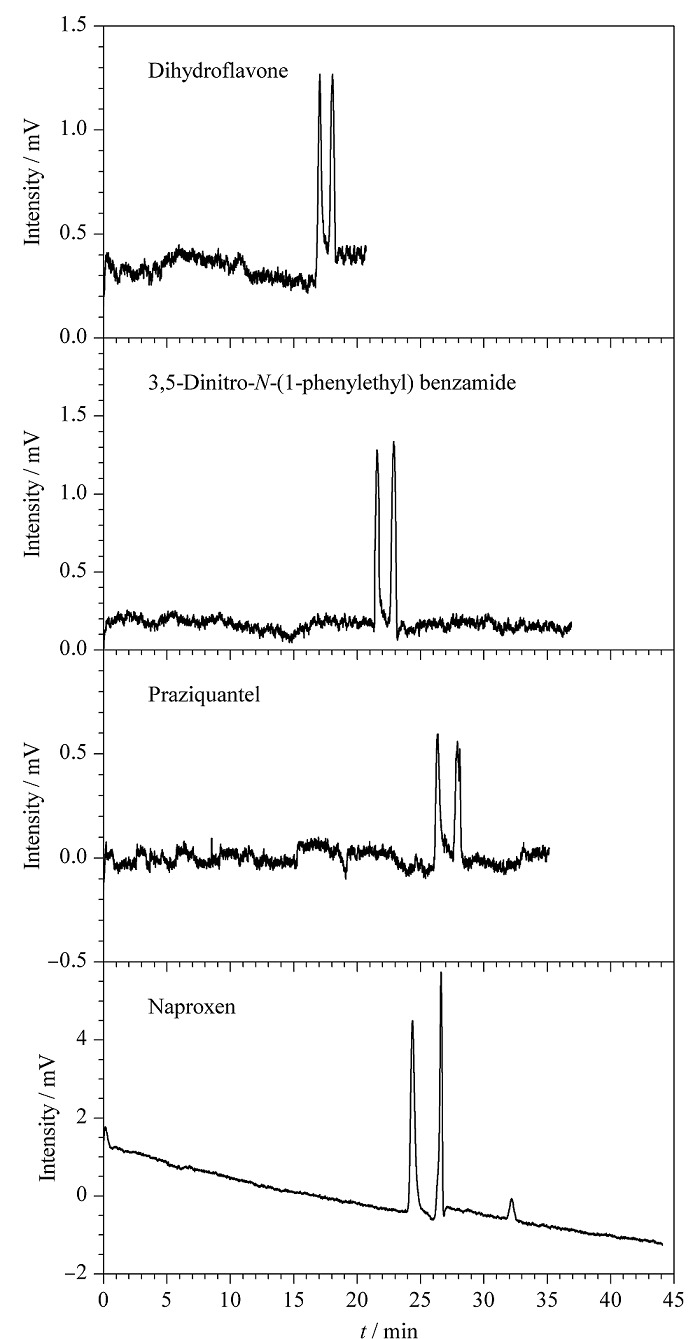
POCs手性柱对4种外消旋体的电色谱图

### 2.6 POCs色谱柱对位置异构体的拆分

POCs毛细管柱对位置异构体*o*,*m*,*p*-碘苯胺、*o*,*m*,*p*-硝基苯胺也进行了分离研究(见图7)。电色谱分离介质为0.100 mol/L、pH 3.51的Tris-H_3_PO_4_缓冲溶液,分离电压为15 kV。位置异构体与POCs固定相间存在着相互作用力,因位置异构体的分子结构、大小各不相同,与固定相接触时产生的效果存在差异,在苯系位置异构体中,其邻、间、对位的长宽比不一样,与固定相的作用力大小也不一样,其保留时间不同,从而使位置异构体分离。

**图7:**
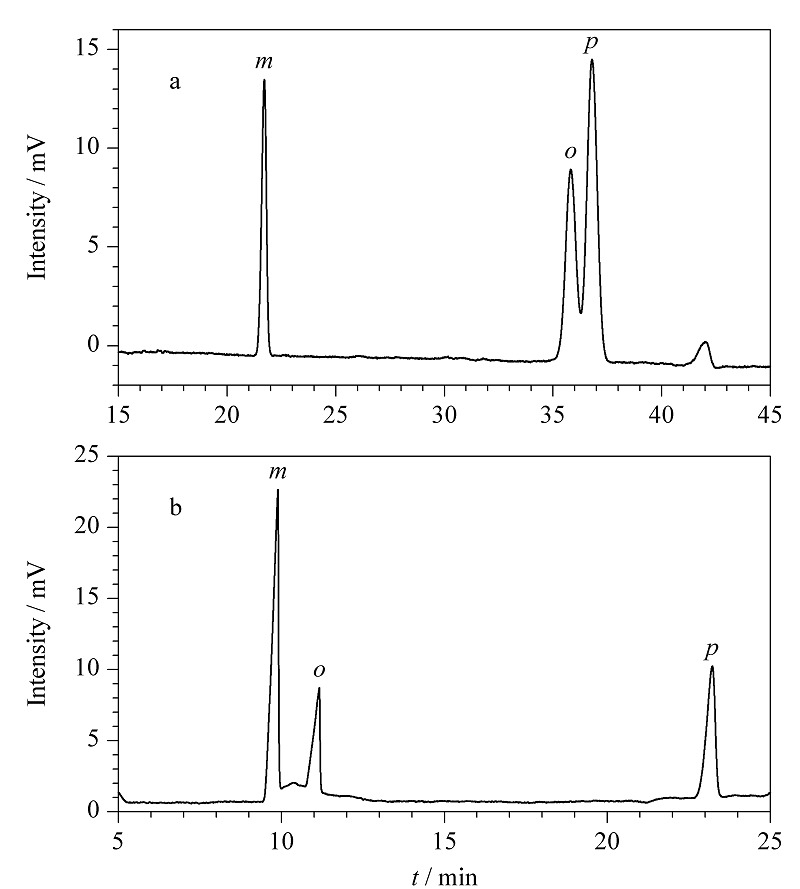
POCs手性柱对(a)*o*,*m*,*p*-硝基苯胺和 (b)*o*,*m*,*p*-碘苯胺的分离电色谱图

## 3 结论

将手性POCs用作毛细管电色谱固定相,能对二氢黄酮、吡喹酮、萘普生、3,5-二硝基-*N*-(1-苯乙基)苯甲酰胺手性化合物进行基线分离,对*o*,*m*,*p*-硝基苯胺、*o*,*m*,*p*-碘苯胺位置异构体具有良好的分离效果。该研究表明,手性POCs是一类很有发展潜力的手性分离材料,对该类材料的深入研究,必将推进手性识别材料的发展,拓展毛细管电色谱在手性分离领域的广泛应用。
